# 3D Nanofibrous Scaffolds for Encapsulation-Controlled Vancomycin Delivery: Antibacterial Performance and Cytocompatibility

**DOI:** 10.3390/polym17233116

**Published:** 2025-11-24

**Authors:** Tatiana Rita de Lima Nascimento, Aline Lima Guérin, Mariana Souza Rodrigues, Camila Félix da Silva, Bruno Martins Maciel, Abdulaziz Alhotan, Saleh Alhijji, Marilia Mattar Amoêdo Campos Velo, Lúcio Roberto Cançado Castellano

**Affiliations:** 1 Institute for Emerging Electronic Technologies (IET), Leibniz Institute for Solid State and Materials Research, 01069 Dresden, Germany; 2 Therapeutic Engineering and Bioproduction in Health Biotechnology (IBIS), Faculty of Sciences, University of Montpellier, 34095 Montpellier, France; 3 Department of Restorative Dentistry, Araraquara School of Dentistry, Sao Paulo State University (UNESP), Araraquara 14801-903, SP, Brazil; 4 Program in Dentistry, Department of Clinical and Social Dentistry, Federal University of Paraiba (UFPB), João Pessoa 58051-900, PB, Brazil; 5 Department of Dental Health, College of Applied Medical Sciences, King Saud University, Riyadh 12372, Saudi Arabia

**Keywords:** vancomycin, nanofibrous scaffolds, PLLA/PEG blend, controlled release, biocompatibility, antimicrobial activity

## Abstract

This study aimed to engineer nanofibrous scaffolds that prioritize architecture, rather than relying solely on the drug, to achieve reproducible, long-acting local therapies. Cotton-wool-like fiber, three-dimensional (3D) poly(L-lactic acid)/polyethene glycol (PLLA/PEG) blend scaffolds were fabricated using solution blow spinning (SBS) as a customizable encapsulation platform for controlled antibiotic release. Morphological and wettability analyses were performed by scanning electron microscopy (SEM) and pendant-drop contact angle measurements, respectively. Fiber diameters were quantified using ImageJ. The chemical composition and thermal behavior were investigated by Fourier-transform infrared spectroscopy (FTIR), differential scanning calorimetry (DSC), and thermogravimetric analysis (TGA). In vitro, assays were conducted to assess the antimicrobial activity of vancomycin-loaded scaffolds against *Staphylococcus aureus* (disk diffusion method), as well as their cytocompatibility (Live/Dead assay in Vero cells) and hemocompatibility (ASTM F756-17 hemolysis test). All biological data were statistically analyzed using ANOVA with Tukey’s post-test, Mann–Whitney, and paired *t*-tests, with significance set at *p* ≤ 0.05. Structural optimization identified PLLA/PEG 85:15 as the most stable composition, producing homogeneous mats with high porosity and rapid wettability. Incorporation of vancomycin (10 wt.%) reduced the fiber diameter (0.23 ± 0.11 µm) compared with unloaded scaffolds (0.32 ± 0.17 µm), indicating drug–polymer interactions that modulated jet elongation. FTIR, DSC, and TGA analyses confirmed polymer miscibility and stabilization of VMC within the fibrous matrix, with no signs of degradation. Drug release exhibited a biphasic profile, with an initial burst during the first 72 h. PLLA/PEG–VMC scaffolds produced larger inhibition zones against *S. aureus* (18.55 mm ± 1.2 to 6.63 mm ± 0.2 at 120 h) compared with free VMC (12.91 mm ± 3.8 to 4.07 mm ± 0.6291), while blank scaffolds were inactive. Hemolysis remained within the range 2% < PLLA/PEG–VMC < 5%, indicating acceptable hemocompatibility according to ASTM standards. Although VCM-loaded PLLA/PEG scaffolds slightly reduced Vero cell viability, no statistically significant differences were observed compared with the control group. These findings demonstrate that the architecture of nanofibers presents itself as a potential platform for antimicrobial therapy with topical vancomycin in potential applications such as wound dressings or implant coatings.

## 1. Introduction

*Staphylococcus aureus* (*S. aureus*) is an opportunistic pathogen that frequently colonizes the nasal cavity and skin. Once host barriers are breached, it becomes a clinically relevant cause of infection with substantial morbidity [1,2,3]. The clinical spectrum ranges from skin and soft tissue infections (e.g., abscesses, cellulitis) to life-threatening conditions such as bacteremia, osteomyelitis, pneumonia, endocarditis, meningitis, and device- or implant-associated infections [2].

Vancomycin (VMC) is a glycopeptide antibiotic that remains a first-line therapy against *S. aureus*, particularly methicillin-resistant strains (MRSA) [4,5,6]. Its bactericidal activity arises from peptidoglycan biosynthesis in Gram-positive organisms [7]. However, widespread use has been associated with the development of resistance, persistent and chronic infections, and a considerable health-economic burden [8]. Intravenous administration is the standard route [9], and the 24-h area-under-the-curve to MIC ratio (AUC24/MIC) is the most reliable PK/PD target for VMC therapy [10]. Systemic delivery, nevertheless, is limited by poor local exposure at infected interfaces, repeated dosing requirements, risks of nephrotoxicity and ototoxicity, and formulation stability constraints [7,11].

Local approaches, such as applying vancomycin powder intraoperatively, can achieve high initial concentrations but are typically followed by rapid drug depletion within one to three days. This can allow recolonization and late infection, highlighting the need for controlled and prolonged local delivery [12]. Degradable polymer nanostructures have emerged as promising platforms for localized, sustained release [8,13]. Among these, cotton-wool-like nanofibers produced by solution blow spinning (SBS) offer scalability, rapid fabrication, high surface area, and minimal material loss [14]. Their multidirectional lay-down generates a porous 3D architecture that promotes drug dispersion, fluid absorption, and gas exchange, while providing the handling familiarity of cotton dressings with the added functionality of a drug reservoir [15].

Recent studies have demonstrated the potential of vancomycin-loaded nanofibers for postoperative and implant-adjacent therapy, including core–shell designs for extended release [16] and hybrid matrices tailored to the infected microenvironment [17]. A persistent challenge, however, is achieving effective antibacterial activity at reduced VMC loadings, in order to mitigate cytotoxicity risks while preserving sustained release [11].

Here, we present cotton-wool-like PLLA–PEG nanofiber bandages fabricated by SBS as a platform for vancomycin encapsulation and controlled local delivery. We hypothesize that encapsulation within PLLA–PEG (i) stabilizes VMC without disrupting polymer crystallinity, (ii) maintains concentrations above the MIC for prolonged periods, and (iii) preserves antibacterial efficacy with minimal hemolysis and acceptable cytocompatibility. Our objective is to demonstrate the effectiveness of PLLA–PEG/VMC bandages as an innovative, biocompatible platform for controlled local antibiotic therapy.

## 2. Materials and Methods

### 2.1. Materials

Poly(L-lactic acid) (PLLA, Mw= 1.25 × 10^5^ g.mol^−1^, intrinsic viscosity 1.8 dL·g^−1^; Polysciences, Warrington, PA, USA); poly(ethylene glycol) (PEG, Mw ≈ 9000 g·mol^−1^; Sigma-Aldrich, Darmstadt, Germany); phosphate-buffered saline (PBS) tablets (Sigma-Aldrich, Darmstadt, Germany); chloroform, 99% (F. Maia, Belo Horizonte, Brazil); vancomycin hydrochloride (ABL Antibiotics of Brazil, São Paulo, Brazil); agar and Brain Heart Infusion (BHI) broth (KASVI, São José dos Pinhais, Brazil); *Staphylococcus aureus* ATCC 15656.

### 2.2. Preparation of PLLA/PEG and Vancomycin-Loaded Solutions

PLLA/PEG blends with weight ratios of 85:15, 70:30, 50:50, and 30:70 (*w*/*w*) were prepared to optimize solution homogeneity. Formulations containing ≥50% PEG produced film-like deposits, likely due to the reduced viscosity associated with the lower molecular weight of PEG. For each composition, 1.0 g of total polymer was dissolved in 10 mL of chloroform (1:10 *w*/*v*, 10 wt.%) under magnetic stirring at 25 ± 2 °C until a clear and homogeneous solution was obtained. Higher-concentration tests (20 wt.%) were prepared by dissolving 2.0 g of polymer in the same solvent volume. Specifically, the PLLA/PEG 85:15 and 70:30 blends contained 0.85/0.15 g and 0.70/0.30 g of PLLA/PEG, respectively.

Previously, to rationalize the antibiotic incorporation, a theoretical equivalence was established with the CLSI/EUCAST diffusion assay standard, in which each 6 mm disc (0.283 cm^2^) contains 30 µg of vancomycin. The 3D fibrous scaffold produced herein (~30 cm^2^) corresponds to approximately 106 standard discs. Incorporation of 0.10 g (100 mg) of vancomycin hydrochloride yields a normalized surface loading of ≈3.33 mg cm^−2^, equivalent to ≈942 µg per 6 mm disc, about 31 times higher than the standard microbiological disc. This enhanced loading reflects the three-dimensional architecture and sustained-release profile of the scaffold, designed to maintain bactericidal concentrations above the minimum inhibitory concentration (MIC) for extended periods. In summary, for a pharmacological context, the typical intravenous dose for adults is 15–20 mg·kg^−1^ (≈1 g per administration), while single doses > 2 g or plasma concentrations >50–80 µg·mL^−1^ are considered potentially toxic. Therefore, the total vancomycin incorporated (100 mg) represents approximately one-tenth of a therapeutic dose and at least twentyfold below the systemic toxic threshold, confirming the local and safe nature of the antibiotic loading.

**Figure 1:**
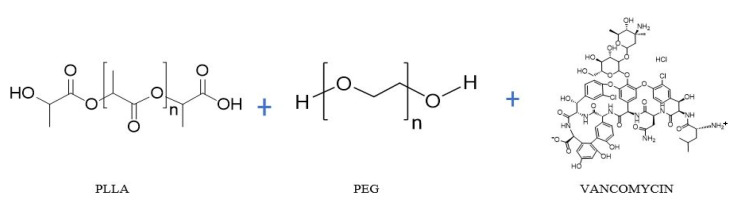
Chemical structures of the PLLA, PEG, and VMC (Generated by the author using the ChemSketch software, ACD/Labs).

Accordingly, for drug-loaded formulations, vancomycin hydrochloride (0.10 g; 10 wt.% relative to total polymer) was pre-dispersed in 1 mL of chloroform via ultrasonication (QSonica Q55 (QSonica, Newtown, CT, USA), 55 W, 2 min) and then added dropwise to the polymer solution under constant stirring until complete homogenization. Stable and transparent PLLA/PEG 85:15 and 70:30 solutions, with and without vancomycin, were obtained and selected for the subsequent spinning process [18,19,20,21,22] Chemical structure of the PLLA, PEC and VMC is shown in Figure 1.

### 2.3. Fabrication of PLLA/PEG and PLLA/PEG–VMC Scaffolds

**Figure 2:**
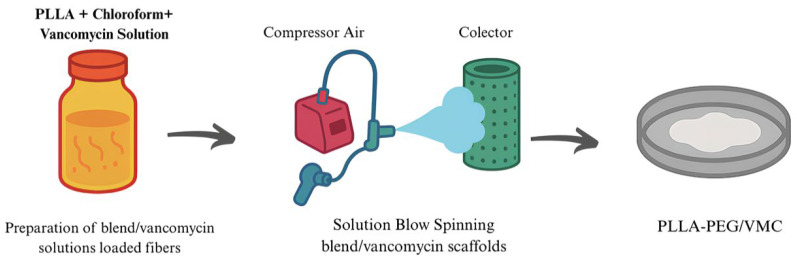
Solution Blow Spinning Process.

The nanofibers were produced using the solution blow spinning (SBS) technique [18,19,20]. A concentric nozzle system composed of three stainless steel tubes (0.8, 1.2, and 3.0 mm in diameter) was used. PLLA/PEG and PLLA/PEG-VMC solutions were injected through the inner tube using syringe pumps (KDS 100 Legacy Syringe Pump), while the outer tube was connected to a compressed air source [21,22]. The optimized processing parameters were air pressure of 10 psi, feed rate of 7.2 mL·h^−1^, nozzle protrusion of 2.5 mm, working distance of 15 cm, and collector rotation speed of 165 rpm (Figure 2). The process was conducted under controlled environmental conditions (33 °C and 50% ± 5% relative humidity).

**Figure 3:**
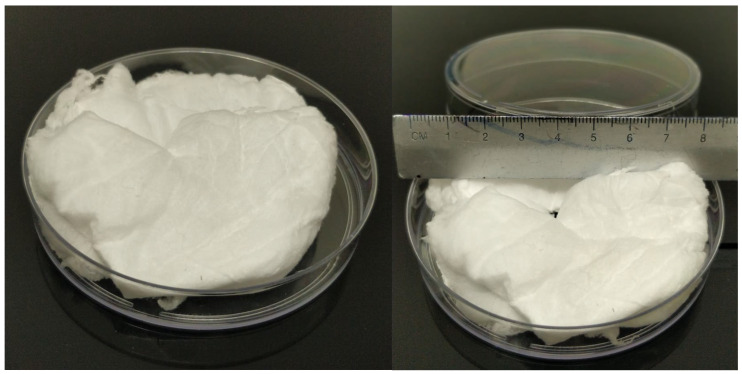
Three-dimensional cotton-like morphology of the PLLA–PEG/VMC scaffolds produced by solution blow spinning.

Under these optimized processing conditions, the total mass yield of nanofibers was determined from the polymer concentration and the solution feed rate. For 10% by weight polymer solutions (1.0 g of polymer in 10 mL of chloroform) supplied at 7.2 mL·h^−1^, the theoretical mass of polymer deposited was approximately 720 mg per hour. This corresponds to 612 mg·h^−1^ of PLLA and 108 mg·h^−1^ of PEG for the 85:15 (*w*/*w*) mixture, and 504 mg·h^−1^ of PLLA and 216 mg·h^−1^ of PEG for the 70:30 (*w*/*w*) mixture. In both cases, the final fibrous mats obtained from a total spinning volume of 10 mL showed uniform coverage over an area of ~30 cm^2^. They resemble a thick cotton-like bandage for wounds, as illustrated in Figure 2 and Figure 3.

### 2.4. Morphology and Wettability

Scanning electron microscopy (SEM; LEO 1430, Zeiss, Oberkochen, Germany) was used to image the 3D bandages. Samples were mounted on aluminum stubs and each sample was sputter-coated with an 8 nm gold layer to prevent surface charging of the non-conductive materials (Gatan Model 682 Precision Etching Coating System, Pleasanton, CA, USA). Fiber diameters were quantified using ImageJ (Version 1.53 t).

The randomly selected fibers were analyzed in accordance with current recommendations for fiber statistics [23]. Wettability was assessed by pendant-drop contact angle. Rectangular specimens (10 mm^2^) were affixed to glass slides (Scotch Magic™ tape, 3M, St. Paul, MN, USA) to obtain flat regions. A 5 µL drop of deionized water was dispensed, and spreading/absorption was recorded continuously (n = 3 per condition) [14,24].

### 2.5. Fourier-Transform Infrared Spectroscopy (FTIR)

PLLA/PEG and PLLA/PEG + VMC scaffolds were examined by FTIR (Shimadzu IRAffinity-1). Spectra were collected in the range 4000–600 cm^−1^ with a resolution of 4 cm^−1^ and 40 scans, using KBr pellets containing 2% (*w*/*w*) of the sample. FTIR assignments were based on reports for PLLA-based scaffolds [20]. Chemical structure of PLA/PEG/Vanco nanofibers Infrared (IR) spectra of the fibers were recorded on an IRAffinity-1/FTIR-8000 spectrometer (Shimadzu, Duisburg, Germany) in reflectance mode at room temperature. All samples were scanned in the region from 4000 to 400 cm^−1^ with a resolution of 4 cm^−1^ [25]. The spectra were taken from an average of 40 scans for each sample, with 3 repetitions per sample [26].

### 2.6. Thermal Analysis (DSC and TGA)

The thermal behavior of PLLA/PEG and PLLA/PEG + VMC scaffolds was investigated using a DSC-60 calorimeter (Shimadzu, Kyoto, Japan). Approximately 6 mg of sample was heated from 25 to 180 °C under a nitrogen atmosphere (50 mL·min^−1^) at a heating rate of 10 °C·min^−1^.

Thermal stability was assessed using a DTG-60H thermogravimetric analyzer (Shimadzu, Kyoto, Japan). About 6 mg of each scaffold was heated from room temperature to 800 °C under nitrogen (50 mL·min^−1^) at a heating rate of 10 °C·min^−1^. Differential scanning calorimetry (DSC) and thermogravimetric analysis (TGA) are commonly used to evaluate polymer miscibility and drug–polymer interactions in nanofiber systems [27].

### 2.7. In Vitro Release of Vancomycin

Drug release was assessed in PBS (0.15 M, pH 7.4) at 37 °C, 140 rpm. Nanofibers (30 mg; ~3 mg theoretical VMC loading) were immersed in 30 mL PBS under sink conditions. At predetermined time points (3, 5, 24, 48, 72, 96, 148, and 172 h), 3 mL of medium was withdrawn and replaced with fresh PBS. UV quantified VMC concentrations–Vis spectroscopy at 280 nm using a calibration curve (UV-2550, Shimadzu, Kyoto, Japan). All measurements were performed in triplicate. Protocols were developed based on current practices for antibiotic-loaded nanofibers [12,28,29].

### 2.8. Antimicrobial Susceptibility

Antibacterial activity was evaluated by agar disk diffusion against *S. aureus* on BHI agar. Inoculation was standardized to 0.5 McFarland (~1.5 × 10^8^ CFU·mL^−1^). Disks of nanofiber mats (5 mm) were placed on agar plates, and 30 µg VMC paper disks were used as positive controls. Plates were incubated at 37 °C for 120 h. Inhibition zones were imaged and quantified using ImageJ. Disk diffusion remains a widely used preliminary screening method for drug-loaded scaffolds [16].

### 2.9. Cytocompatibility and Hemocompatibility

#### 2.9.1. Vero Cell Viability (Live/Dead Assay)

Cell viability was also determined using the Live/Dead Cytotoxicity Kit for Mammalian Cells (Molecular Probes, Eugene, OR, USA). Initially, cells were cultured in 24-well plates, in triplicate (n = 3), at a density of 2 × 10^4^ cells. After 24 h of treatment for each group (Control, PLLA-PEG, PLLA-PEG-VERO), the culture medium was removed, and the cells were incubated with 2 μM calcein AM and 4 μM ethidium homodimer. The wells were then washed with PBS and observed under a fluorescence microscope (Eclipse Ti-U, Nikon Instruments, Melville, NY, USA) using excitation filters at 485 nm and 530 nm. For each sample, images of three random fields were acquired and subsequently processed using the NIS-Elements software (Nikon Instruments, Melville, NY, USA). The number of live and dead cells in each field was then counted, and the ratio and percentage of live and dead cells in each experimental group were calculated. Cell viability was quantified according to the following equation:

$$\mathrm{Cell}\;\mathrm{viability}\;(\%)=\frac{N_{v}\times 100}{\bar{N_{v,control}}}$$

where $N_{v}$ represents the number of viable cells in each experimental condition, and $\bar{N_{v,control}}$ denotes the mean number of viable cells in the control group.

#### 2.9.2. Hemolysis

A hemolysis test was performed according to ASTM F756-17 [30] to measure the impact of erythrocyte lysis that the PLLA-PEG-VCM and PLLA-PEG scaffolds can produce. A trained health worker took 3 mL of a blood sample from three healthy donors into a heparin tube utilizing venipuncture (Becton Dickinson, Franklin Lakes, NJ, USA). It is important to note that biosecurity parameters were considered. Erythrocytes were separated by centrifugation (3000 rpm for 4 min) and washed thrice with PBS solution. A final solution of 1% *v*/*v* of erythrocytes in PBS was prepared. Moreover, each scaffold’s circular pieces (6 mm diameter) were sterilized with UV light exposition for 15 min on each side. The scaffolds were incubated in microtubes, by quadruplicate, in 200 μL of the 1% *v*/*v* erythrocytes solution for 30 min, at 37 °C and 400 rpm. An erythrocytes and PBS solution (1% *v*/*v*) was taken as a negative control, not exposed to films. An erythrocytes solution in distilled water (1% *v*/*v*) was taken as a positive control. After incubating samples and controls, tubes were centrifuged at 14,000 rpm for 3 min. Hemoglobin released in the supernatant was measured by UV–Vis spectroscopy (Nanodrop, Thermo Fisher Scientific, Waltham, MA, USA) at 415 nm. The percentage of hemolysis was obtained with Equation (1).

(1) $$\%\;of\;Hemolysis=\frac{A_{Sample}-A_{Negative}}{A_{Positive}-A_{Negative}}\;\times 100$$

The hemocompatibility testing for the drug-loaded assay was performed by adapting the instructions described in [31]. Positive control: distilled water; negative control: physiological saline. The <2% is non-hemolytic, and <5% acceptable.

### 2.10. Statistical Analysis

All biological data were analyzed using GraphPad Prism 8.0 (GraphPad Software Inc., San Diego, CA, USA). For Vero cell cytotoxicity assay, the Shapiro–Wilk test was applied to assess the normality of data distribution, which confirmed that the data followed a normal distribution. Consequently, a one-way ANOVA parametric test was performed for intergroup comparison followed by Tukey’s multiple comparisons post-test. The non-parametric Mann–Whitney U test was used to determine statistical differences in the disk diffusion assays, while paired *t*-tests were applied to analyze hemolysis results. Statistical significance was considered at *p* ≤ 0.05 (95% confidence level).

## 3. Results

### 3.1. Analysis of Antibacterial Scaffolds

The PLLA/PEG blends were successfully prepared, with the 85:15 composition identified as the most stable for SBS processing. At this ratio, continuous and homogeneous nanofibers were produced, whereas blends containing more than 30% PEG showed impaired fiber formation, low yield, and poor detachment from the collector. These limitations are likely to be due to decreased solution viscosity and increased blend plasticity. Vancomycin was incorporated at 10 wt.% based on its minimum inhibitory concentration (MIC, 15 µg·mL^−1^) against *S. aureus*. VMC-loaded bandages were produced reproducibly (Figure 3).

### 3.2. Morphology, Surface Area and Chemical and Thermal Characterization

**Figure 4:**
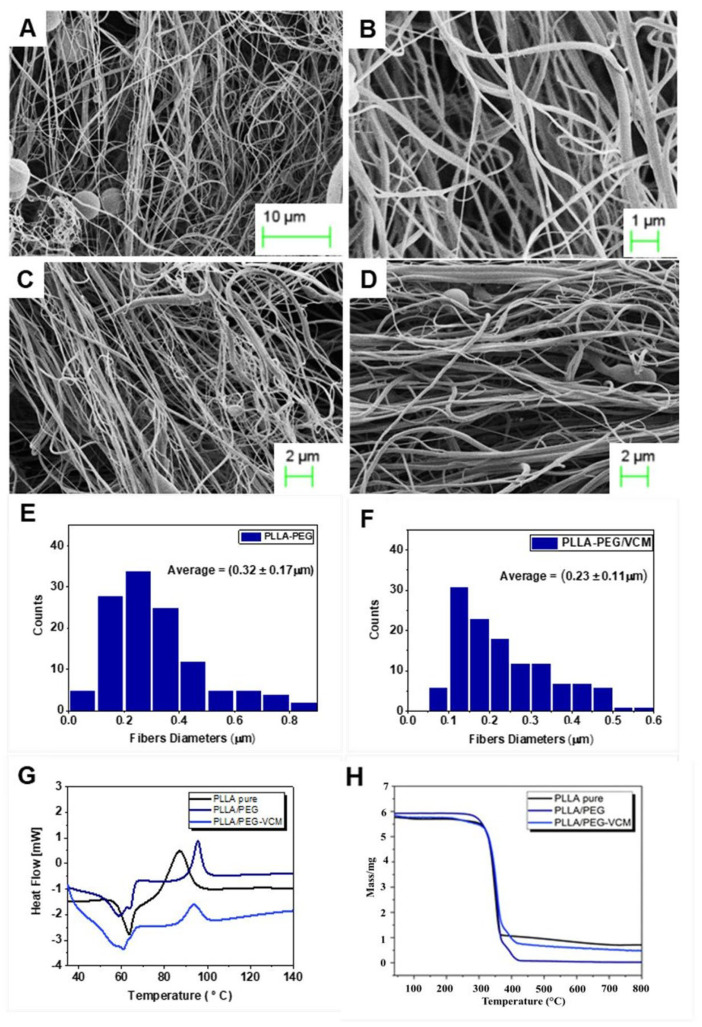
SEM characterization of nanofibers. (**A**) Surface morphology of PLLA–PEG. (**B**) Fiber of PLLA–PEG. (**C**) Surface morphology of PLLA–PEG/VMC. (**D**) Fiber of PLLA–PEG/VMC. (**E**) and (**F**) Fiber diameter. (**G**) DSC curves of control PLLA, PLLA–PEG, and PLLA–PEG/VMC scaffolds. (**H**) TGA curves of control PLLA, PLLA–PEG, and PLLA–PEG/VMC scaffolds.

Scanning Electron Microscopy (SEM) analysis revealed that the PLLA/PEG (85:15) scaffolds were primarily composed of continuous nanofibers interspersed with occasional isolated granules, exhibiting an average fiber diameter of 0.32 ± 0.17 µm. The incorporation of VMC led to a significant reduction in the mean fiber diameter to 0.23 ± 0.11 µm. Comparable decreases in fiber dimensions have been documented in previous studies on nanofibrous systems and are generally attributed to drug–polymer interactions that compromise solution homogeneity and influence jet elongation dynamics during the electrospinning process [14]. Representative morphologies are presented in Figure 4.

Wettability testing demonstrated that PLLA/PEG 85:15 scaffolds absorbed water droplets within 3 s in all specimens, with an initial mean contact angle of 45° ± 2°. The incorporation of antibiotic further enhanced hydrophilicity, facilitating rapid fluid absorption, a desirable property for wound-healing applications [32,33].

DSC analysis (Figure 4G) indicated that the PLLA/PEG blends were miscible, as evidenced by shifts in the glass transition temperature (Tg) with increasing PEG incorporation. The presence of VMC slightly modified the thermal transitions, which can be attributed to hydrogen bonding via the hydroxyl groups of vancomycin. TGA curves (Figure 4H) showed major degradation events at approximately 400 °C, with an additional shoulder at 360–370 °C corresponding to PEG content. Importantly, no premature degradation of VMC was detected, suggesting stabilization of this otherwise labile drug within the fibrous matrix [17].

### 3.3. FTIR Analysis of Nanofibrous Scaffolds

**Figure 5:**
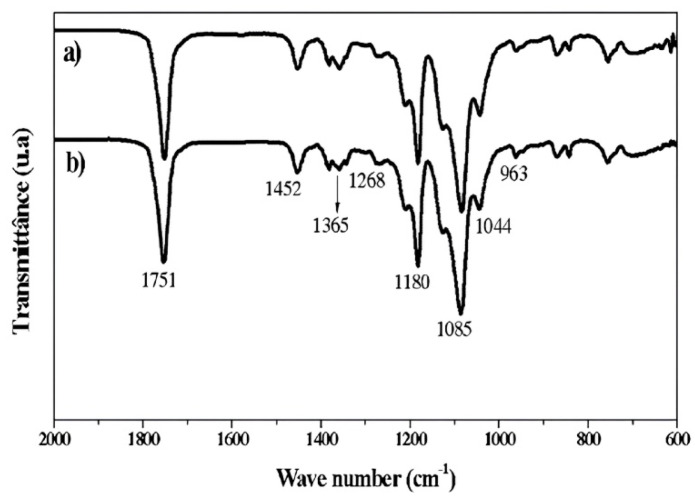
The transmission FTIR spectra shows the characteristic bond vibrations of the scaffolds. In (**a**), the spectral profile of PLLA–PEG is presented, while in (**b**) the spectrum of PLLA–PEG/VMC is shown. The accompanying table summarizes the main detected peaks, indicating the corresponding functional groups and their respective vibrational modes.

The infrared spectra of the fibers (Figure 5) exhibited two intense bands at 1751 cm^−1^ and 1085 cm^−1^, corresponding to ester carbonyl (C=O) stretching and C–O stretching vibrations of the PLA backbone, respectively. Additional bands were detected at 1452 cm^−1^ and 1365 cm^−1^, attributed to asymmetric and symmetric bending modes of C–H bonds. A weaker band at 1268 cm^−1^ was assigned to C–H stretching, while absorptions at 1180 cm^−1^ and 1044 cm^−1^ reflected symmetric and asymmetric stretching of C–O and C–O–C groups, characteristic of aliphatic polyesters. Peaks at 963, 872, and 755 cm^−1^ were associated with C–COO symmetric stretching. These findings are consistent with previous reports describing the FTIR profiles of PLLA and drug-loaded PLLA/PEG nanofibers, where the preservation of the main absorption bands indicates that vancomycin encapsulation did not disrupt the polymer backbone. Minor spectral shifts observed in VMC-loaded scaffolds can be attributed to weak hydrogen-bonding interactions between the drug and the polymer matrix, as reported similarly for antibiotic-incorporated nanofibers [17].

### 3.4. Release Scaffold Availability

**Figure 6:**
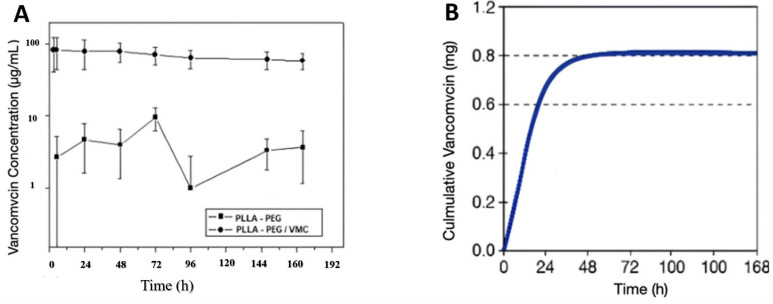
Released profile and cumulative quantification of vancomycin (VMC) from PLLA-PEG scaffolds. (**A**) VMC concentration as a function of time showing a biphasic released pattern characterized by an initial burst during the first 72 h (~100 μg mL^−1^), followed by a sustained release above 80 μg mL^−1^ up to 168 h. Control scaffolds (PLLA-PEG without VMC) exhibited negligible release (3–10 µg mL^−1^). (**B**) Cumulative vancomycin release indicating that approximately 80–100% of the theoretical loading (~0.6 mg VMC per 6 mg scaffold patch) was released over 7 days.

The release profile of VMC from PLLA/PEG scaffolds (Figure 6A) exhibited a biphasic pattern. An initial burst during the first 72 h yielded VMC concentrations of approximately 100 µg·mL^−1^, followed by sustained release above 80 µg·mL^−1^ for up to 168 h. Control scaffolds without VMC exhibited negligible release (3–10 µg·mL^−1^), which was well below MIC levels. Based on the theoretical loading (~0.6 mg VMC per 6 mg scaffold patch) (Figure 6B), the cumulative release corresponded to 80–100% of the expected payload. These findings are in line with previous studies reporting biphasic antibiotic release from electrospun or blow-spun fibers, where an initial burst ensures rapid antibacterial action and sustained delivery maintains therapeutic concentrations [34,35]. Notably, encapsulation within PLLA/PEG nanofibers stabilized vancomycin, preventing premature degradation and ensuring therapeutic bioavailability.

### 3.5. Antibacterial Activity, and Biocompatibility

**Figure 7:**
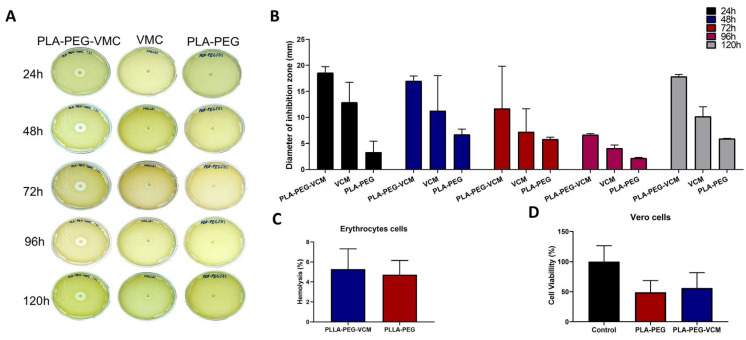
PLLA/PEG + VCM scaffold biological activity. (**A**) Antibacterial activity against *S. aureus* by disk diffusion showing inhibition zones up to 120 h. (**B**) Mean value of the diameter of the zones of inhibition (mm) over time for PLLA–PEG-VMC, free VMC, and PLLA–PEG. (**C**) Hemolysis percentage for PLLA–PEG and PLLA–PEG/VMC scaffolds (mean ± SD, n = 3). (**D**) Quantitative evaluation of groups to live cell percentage of Vero cell line. All data represents three independent experiments and are expressed as mean ± SD.

Disk diffusion assays confirmed that PLLA/PEG + VMC scaffolds produced inhibition zones ranging from 18.55 mm (±1.2) to 6.63 mm (±0.2) against *S. aureus* after 120 h (Figure 7B). Free vancomycin disks generated halos ranging from 12.91 mm to 4.07 mm under the same conditions, while unloaded scaffolds exhibited no antimicrobial activity. Comparing the differences among the groups, no statistical significance was observed.

Hemolysis tests (Figure 7C) showed that PLLA/PEG + VCM and PLLA/PEG scaffolds exceeded the 2% hemolysis value but still presented acceptable levels (<5%), in accordance with the biocompatibility threshold recommended by ASTM F756-17 [30]. No statistically significant differences were observed between groups.

Cytocompatibility was further assessed in Vero cells (Figure 7D) through the live/dead assay. The Vero cell line showed a decreased percentage of viable cells when exposed to PLLA/PEG + VMC and PLLA/PEG scaffolds, with viability rates of 56% and 48.8%, respectively. In the control comparison among the groups, no significant reduction in cell viability percentage was observed: control vs. PLLA/PEG + VMC (*p* = 0.0881) and control vs. PLLA/PEG (*p* = 0.1418).

The resulting cell viability percentages were calculated for all experimental conditions and expressed as mean ± standard deviation (SD) (Figure 7). No statistically significant differences were observed among the experimental groups (p>0.05).

## 4. Discussion

*Staphylococcus aureus* remains a significant cause of pyogenic infections, readily colonizing skin and mucosal surfaces and penetrating deeper tissues, where it can lead to complications such as bacteremia, osteomyelitis, endocarditis, and implant-associated infections. In the context of implant rehabilitation, *S. aureus* colonization and biofilm formation on materials such as titanium are key contributors to osseointegration failure and chronic infection [15,36].

Strategies that deliver antimicrobials locally and prevent early colonization are therefore highly desirable. In this study, we developed vancomycin-loaded PLLA/PEG scaffolds produced by solution blow spinning to provide sustained antibiotic release with minimal cytotoxic effects. A vancomycin loading of 10 wt.% was selected based on its MIC (15 µg·mL^−1^) against *S. aureus* and previous reports applying MIC multiples in fibrous devices. Although systemic vancomycin dosing (1 g every 12 h) is vastly higher, our scaffold design enables high local concentrations while limiting systemic exposure [17,37,38,39].

The incorporation of PEG and vancomycin into PLLA altered scaffold wettability. While plain PLLA tends to exhibit hydrophobic behavior, possibly linked to the Cassie–Baxter state [40,41], VMC-loaded scaffolds absorbed water instantaneously, reflecting a shift towards hydrophilicity. Morphologically, the VMC-loaded scaffolds exhibited a uniform and predominantly continuous fibrous structure, with isolated and infrequent beads. Such sporadic formations are commonly observed in mats produced by SBS, due to the high-shear airflow characteristic of this technique [42]. These defects, however, do not compromise the material’s performance [42], as its antibacterial activity and drug release behavior are primarily determined by the nanofibrous network as a whole. The observed reduction in fiber diameter following drug inclusion is consistent with the literature, which reports drug–polymer interactions that influence jet dynamics during fiber formation [43]. The nanofibrous architecture not only provides a physical barrier against bacterial infiltration but also enhances antibacterial effectiveness by increasing pathogen contact with the released drug.

The release profile exhibited a biphasic pattern, characterized by an initial burst during the first 72 h. This dual-phase release is in line with previous studies of antibiotic-loaded nanofibers, where early bacterial suppression is coupled with prolonged inhibition [17]. Encapsulation also stabilized vancomycin, protecting it from premature degradation.

Although no dedicated chemical stability assay was performed herein, previous work has demonstrated that vancomycin remains chemically stable (>90% of initial concentration) in aqueous media stored at –20 °C for 90 days and subsequently at 5 °C for 30 days [44].

In the present study, complementary physicochemical analyses (FTIR, DSC, and TGA) showed no detectable changes in the characteristic peaks of either PLLA/PEG or vancomycin, indicating that no degradation or chemical interaction occurred during scaffold preparation or incubation. Therefore, the drug release behavior observed can be attributed mainly to the scaffold’s structural architecture rather than to any decomposition or instability of the incorporated drug [44]

Functionally, disk diffusion assays confirmed that PLLA/PEG–VMC scaffolds reduced the *S. aureus* growth showing a larger inhibition zones than free vancomycin, underscoring the benefits of sustained local delivery. The enhanced efficacy of drug-loaded scaffolds is consistent with previous reports, Pérez-Davila et al. (2023) [45] showed that in his three methods to incorporate VCM (Dip coating, Drop coating, and print 3D) the antibacterial was effective against *S. aureus*. Drip and drop coating showed a major inhibition halos (~27–28 mm) than the present study, while 3D printing showed a minor halo than our work (~16 mm).

Biocompatibility assays further confirmed the suitability of the scaffolds. Hemolysis tests revealed that both VCM-loaded and unloaded scaffolds exhibited hemolytic rates above 2%. This assay is widely used because red blood cells are highly sensitive; their membranes rupture when exposed to incompatible materials. Upon lysis, hemoglobin is released into the medium, allowing quantification through changes in optical density. According to F756-08, and F756-17 (Standard Practice for Assessment of Hemolytic Properties of Materials) the hemolytic properties of biomaterials are classified based on the percentage of released hemoglobin (% rHb) after incubation with blood: 0–2% rHb indicates non-hemolytic, 2–5% rHb indicates slightly hemolytic, and >5% rHb indicates hemolytic behavior [31]. Therefore, all tested scaffolds can be considered slightly hemolytic and biocompatible with blood components.

Additionally, cytotoxicity results in Vero cells showed that both VCM-loaded and unloaded scaffolds reduced cell viability to 56% and 48.8%, respectively, with no significant differences compared to the control group. Our findings diverged from those reported by Pérez-Davila et al. (2023) [45], who observed cell viability above 90% for the NCTC clone 929 cell line (ECACC 88102702, mouse fibroblasts) and for MG63 cells (osteoblast-like cells) after direct incubation for 6 days on the three types of vancomycin-loaded scaffolds, indicating that drug loading did not affect viability. These divergent results may be related to the specific characteristics of the materials used in each study and to differences in the cell lines employed.

Similarly, Naeimi et al. (2020) [46] incorporated vancomycin into chitosan/PVA/PEG hydrogels and reported inhibition zones of 19–22 mm against *S. aureus*. In their cytotoxicity assays, vancomycin-loaded hydrogels (PVA/CS and PVA/CS/PEG) exhibited cell viability comparable to the control in L929 fibroblasts after 48 h of incubation. These findings further support the antibacterial efficacy and biocompatibility of vancomycin-loaded polymeric systems; however, variations in material composition and cell models may explain the different biological responses observed across studies.

The correlation between the estimated amount of vancomycin (VMC) incorporated and the biological performance of the fibrous scaffolds can corroborate the efficiency of the controlled release mechanism. Although the total amount of incorporated VMC (≈100 mg per 30 cm^2^ scaffold) was substantially higher than that used in standard diffusion discs, the slow and sustained release from the nanofibrous architecture ensured that only a fraction of the drug was released at any given time. This gradual diffusion maintained bactericidal concentrations on the material surface, as evidenced by the pronounced inhibition zones, while preventing the rapid and massive release typically associated with cytotoxicity. Therefore, despite the higher total antibiotic content, the scaffolds preserved the viability of Vero cells, indicating that the polymeric layer effectively modulated VMC release and mitigated potential toxicity. These results demonstrate that the mass of antibiotic incorporated into the fibers directly correlates with the amount released and the antibacterial efficacy, but the controlled release profile inherent in the 3D structure provided a balanced therapeutic response, maximizing antibacterial performance while maintaining biocompatibility.

Taking together, the results demonstrate that this scaffold design achieves a favorable balance between localized antimicrobial efficacy and mammalian cell compatibility, positioning it as a promising candidate for wound dressings or implant coatings in settings at risk of *S. aureus* infection.

## 5. Conclusions

This study demonstrated that PLLA/PEG nanofibrous scaffolds fabricated by solution blow spinning can serve as effective carriers for vancomycin encapsulation and controlled release. Encapsulation stabilized the labile vancomycin molecule, ensuring a biphasic release profile with an initial burst. Such a release profile is crucial for maintaining therapeutic concentrations while minimizing the cytotoxic risks associated with high systemic dosing.

Physicochemical analyses (FTIR, DSC, TGA) confirmed that vancomycin incorporation did not compromise the structural integrity of the PLLA/PEG matrix. At the same time, morphological characterization revealed uniform, bead-free fibres with enhanced wettability. Antimicrobial assays showed that encapsulated vancomycin retained potent bactericidal activity against *S. aureus*, producing larger inhibition zones than the free drug. Cytotoxicity and hemocompatibility testing confirmed that the scaffolds preserved cell viability and induced hemolysis above 2% but below 5%, highlighting their favorable safety profile.

Overall, these findings provide strong evidence that 3D fibrous scaffolds represent a technically superior platform compared with free-drug administration or non-encapsulated systems. By combining high surface area, tunable porosity, and controlled release profile, such nanofibrous architectures hold significant potential for translation into wound dressings, implant coatings, and other biomedical applications requiring localized antimicrobial therapy.

## Data Availability

The original contributions presented in this study are included in the article. Further inquiries can be directed to the corresponding author.
